# Rare Case of Methemoglobinemia Complicating Pregnancy

**DOI:** 10.1155/2016/9740403

**Published:** 2016-01-04

**Authors:** S. Verma, P. Sachdeva, G. Gandhi

**Affiliations:** Maulana Azad Medical College, Delhi 110002, India

## Abstract

A patient at 38 weeks of gestation when taken for emergency cesarean section had her oxygen saturation of 66 to 70% (by saturation probe on monitor) in operation theatre. She was otherwise asymptomatic but her oxygen saturation was persistently low on the monitors. Her arterial blood gas analysis showed all parameters to be normal. Her electrocardiography was normal. Her surgery was imperative but due to her reduced oxygen saturation she became a high-risk case. In presence of senior consultants of anesthesia and gynecology and under high-risk consent she had an uneventful cesarean delivery. Physician and cardiologist opinions were sought thereafter. The outcomes and the results of our efforts to find the etiology of her reduced saturation on monitors despite being clinically asymptomatic lead to the disclosure of this rare hemoglobinopathy. Mother and baby had uneventful course after delivery and were discharged well.

## 1. Introduction

Methemoglobinemia is a form of hemoglobinopathy, which is characterized by the presence of higher than normal level of methemoglobin. In methemoglobin it is the ferric (Fe^3+^) form of iron, which is attached to globin instead of the ferrous (Fe^2+^) form. Due to this modification hemoglobin has decreased ability to bind with oxygen whereas there is an increased affinity of oxygen to the three other heme sites (that are still ferrous) within the same tetrameric hemoglobin unit. This leads to an overall reduced ability of the red blood cell to release oxygen to tissues; hence oxygen-hemoglobin dissociation curve is shifted to the left.

Normally, methemoglobin levels are <1% in blood. Elevated levels can be caused by reduction or disruption of protective enzyme systems (like cytochrome b5 reductase, diaphorase I, etc.). Excess amount of methemoglobin can cause tissue hypoxia and hence manifest as cyanosis [1]. Broadly they are classified into 2 types: congenital or hereditary and acquired methemoglobinemia. Acquired methemoglobinemia caused by environmental oxidizing agents is common; congenital form is rare [2]. Hereditary methemoglobinemia is caused by deficiency of NADH methemoglobin reductase enzyme (mostly) and is an autosomal recessive disorder. Orphanet put it in the list of rare diseases [3]. A variant of congenital methemoglobinemia (NADH-CYB5R deficiency) is a very rare autosomal recessive disorder [4]. The exact prevalence of hereditary methemoglobinemia is unknown as there are very few cases reported in the literature. Recessive hereditary methemoglobinemia further has two clinical types: type I (in which cyanosis is the only clinical sign) and type II (permanent mild cyanosis is associated with severe neurological manifestations) [5].

## 2. Case Summary

22-year, booked patient in her second pregnancy (previous vaginal delivery) presented to gynaecology emergency at 38-week period of gestation with labour pains and anemia. She was previously admitted with us at 25 weeks of gestation, for evaluation and treatment of severe anemia (hemoglobin of 5.5 g/dL then). In that admission she had complaints of easy fatigability and weakness. After evaluation she then received a unit of blood and the rest of the treatment was continued with parenteral iron therapy (as peripheral smear showed microcytic hypochromic anemia); on discharge her hemoglobin levels improved (9 g/dL) and she was symptomatically better. She could not be followed up later due to lack of her further antenatal visits and noncompliance. Now she came back to us at 38 weeks at late night with labor pains. She was admitted in labor room and all her baseline investigations were sent. She clinically looked pale but had no cyanosis or respiratory discomfort. She had adequate uterine contractions and was monitored for progress of labor. We found her to have poor progress of labor despite adequate contractions and so she was prepared for emergency cesarean section. Her preoperative investigations showed moderate anemia (8.2 g/dL) with normal platelets and normal coagulation profile. After anesthesia clearance patient was taken in operation theatre for procedure.

In operation theatre during her vitals and oxygen saturation monitoring, the oxygen saturation probe showed readings between 66 and 70% in room air. Despite change of saturation probe and removing all artifacts this finding was persistent on the monitor. The patient despite these readings was comfortable with no breathlessness, no palpitations, and no chest pain (absolutely asymptomatic) and on examination no significant cardiovascular and respiratory system findings were found. Her vitals were normal. We immediately sent for arterial blood gas (ABG) analysis from right radial artery, although color of blood was abnormally dark; even on repeat sampling reports were normal (sO_2_ was normal). Absence of cyanosis and disparity between clinical picture of the patient and oxygen saturation on monitors alerted us and we did further evaluation prior to surgery. Electrocardiograph (ECG) was recorded, which was normal. Physician referral was sought and on call senior resident examined the patient. No significant change in saturation status was noticed even with high flow oxygen by mask. So under high-risk consent of postop mechanical ventilation and need of ICU, with anesthesia consultant on floor, she was given spinal anesthesia (2 mL bupivacaine given in sitting position by 25 G spinal needle in L3-L4 space), which was later converted to general anesthesia (induced with 100% oxygen on Bains). Her saturation was maintained at 72%. Cesarean section was uneventful with a newborn of good APGAR. After baby delivery, intraoperatively repeat ABG was sent, which was also normal. Postoperatively, patient was shifted to Intensive Care Unit on ventilation; the medicine team on call reviewed her there. Cardiology opinion was also sought and no underlying cardiac pathology was found. Physicians advised for anemia correction and to evaluate for hemoglobinopathies. She was given three-unit packed cell and continued on intravenous antibiotics. She was off ventilator after 48 hours. Despite her saturation being maintained at 65–72%, patient was asymptomatic and had normal ABG; this abnormal saturation was the reason for suspicion of hemoglobinopathy. Her blood samples were sent to higher laboratory outside our hospital for testing which revealed methemoglobinemia. No active intervention was advised by physicians for her condition as she was presently asymptomatic. She was discharged on the 8th postoperative day with no complications and healthy baby. Her baby was also evaluated though being asymptomatic and found normal. As this condition has genetic predisposition, the baby was subjected to evaluation.

## 3. Discussion

Methemoglobinemia may be congenital or acquired [6]. In the congenital Homozygote's variety, the defect in methemoglobin reductase lacks the ability to convert methemoglobin to hemoglobin and these individuals are cyanosed from birth. But, in heterozygote's variety, there is partial defect in the enzyme; methemoglobinemia may develop when challenged with oxidative drugs [6]. There is another variant of this disorder, which is rare, HbM-Iwate, formed due to the point mutation of alpha globin gene [7]. Hereditary methemoglobinemia is an autosomal recessive disorder and hence is rare.

Acquired methemoglobinemia may result from oxidant drugs including nitrites, nitrates (from contaminated water), aniline dyes, antimalarial drugs, and phenacetin. As the presence of methemoglobin shifts the oxygen dissociation curve to the left, resulting in decreased unloading of oxygen to the tissues, symptoms depend on quantity of methemoglobin [8]. Normal adults can have methemoglobin up to 2% in the blood and its production by autooxidation is balanced via methemoglobin reductase. Clinically detectable cyanosis usually develops at levels over 10%, whereas, at 30%, hypoxia appears and levels of 60–70% are often fatal [6]. Treatment of methemoglobinemia with ascorbic acid or methylene blue is effective [9]. Chronic mild methemoglobinemia may be completely asymptomatic and necessitate no specific therapy. As pregnancy is a stressful event, methemoglobin level can be increased during pregnancy especially during delivery and it may be normalized after delivery [10]. In early pregnancy it can be treated with methylene blue and vitamin C supplements. In this case it was diagnosed postoperatively as methemoglobinemia and as patient was asymptomatic no specific therapy was started.
